# Potential of miRNA-Based Nanotherapeutics for Uveal Melanoma

**DOI:** 10.3390/cancers13205192

**Published:** 2021-10-16

**Authors:** Chun Yang, Rui Wang, Pierre Hardy

**Affiliations:** 1 Research Center of CHU Sainte-Justine, University of Montréal, Montréal, QC H3T 1C5, Canada; chun.yang.hsj@ssss.gouv.qc.ca; 2 Department of Pharmacology and Physiology, University of Montréal, Montréal, QC H3T 1C5, Canada; rui.wang.1@umontreal.ca; 3 Department of Pediatrics, University of Montréal, Montréal, QC H3T 1C5, Canada

**Keywords:** uveal melanoma (UM), microRNA (miRNA), preclinical study, nanodelivery systems, nanoparticles, multifunctional nanoparticles

## Abstract

**Simple Summary:**

Human uveal melanoma (UM) is the most common primary intraocular tumor with high metastatic risk in adults. Currently, no effective treatment is available for metastatic UM; therefore, new therapeutic approaches are needed to improve overall survival. Given the increased understanding of microRNAs (miRNAs) and their roles in UM tumorigenesis and metastasis, miRNA-based therapy may offer the hope of improving therapeutic outcomes. This review summarizes the actions of select miRNAs examined in preclinical studies using miRNAs as therapeutic targets in UM. The focus of this review is the application of established nanotechnology-assisted delivery systems to overcome the limitations of therapeutic miRNAs. A blend of therapeutic miRNAs and nanodelivery systems may facilitate the translation of miRNA therapies to clinical settings.

**Abstract:**

Uveal melanoma (UM) is the most common adult intraocular cancer, and metastatic UM remains deadly and incurable. UM is a complex disease associated with the deregulation of numerous genes and redundant intracellular signaling pathways. As understanding of epigenetic dysregulation in the oncogenesis of UM has increased, the abnormal expression of microRNAs (miRNAs) has been found to be an epigenetic mechanism underlying UM tumorigenesis. A growing number of miRNAs are being found to be associated with aberrant signaling pathways in UM, and some have been investigated and functionally characterized in preclinical settings. This review summarizes the miRNAs with promising therapeutic potential for UM treatment, paying special attention to the therapeutic miRNAs (miRNA mimics or inhibitors) used to restore dysregulated miRNAs to their normal levels. However, several physical and physiological limitations associated with therapeutic miRNAs have prevented their translation to cancer therapeutics. With the advent of nanotechnology delivery systems, the development of effective targeted therapies for patients with UM has received great attention. Therefore, this review provides an overview of the use of nanotechnology drug delivery systems, particularly nanocarriers that can be loaded with therapeutic miRNAs for effective delivery into target cells. The development of miRNA-based therapeutics with nanotechnology-based delivery systems may overcome the barriers of therapeutic miRNAs, thereby enabling their translation to therapeutics, enabling more effective targeting of UM cells and consequently improving therapeutic outcomes.

## 1. Introduction

Uveal melanoma (UM) is the most common adult intraocular malignment tumor arising from melanocytes in the uveal tract, including the iris, ciliary body and choroid. Most UM is found in the choroid (~85%) [1,2,3]. Approximately 50% of patients with UM develop metastasis, most commonly to the liver (89% of metastatic UM patients) [4]. To date, no effective standard treatment is available, and the median survival time of patients with metastatic UM (mUM) is approximately 6–12 months after diagnosis [5,6]. Genetic or phenotypic predisposition, age, sex, work environment and dermatological conditions are risk factors associated with UM, and older patients with UM have a poorer prognosis [7]. Genetic analysis has indicated that UM metastasis and shorter survival is associated with the loss of a copy of chromosome 3 (monosomy 3) and germline mutations of the tumor suppressor gene BRCA-associated protein 1 (BAP1). BAP1 mutations are found in approximately 84% of metastasized UM [8,9,10,11]. Furthermore, a large majority of blue nevi and up to 90% of all UM tumors bear a mutation in the G protein subunit alpha (GNAQ) and subunit alpha-11 (GNA11) genes. Mutations in GNAQ/11 genes are considered an early event or initiating event in UM tumorigenesis and do not increase the risk of metastasis [12]. GNAQ/11 mutations constitutively activate several tumorigenic signaling pathways [13,14]; thus, drugs targeting GNAQ/11 mutations or interfering with critical downstream effectors might be effective in the majority of UM. The driver mutations of eukaryotic translation initiation factor 1A X-linked (EIF1AX), splicing factor 3B subunit 1 (SF3B1), phospholipase C4 or G-protein-coupled receptor cysteinyl leukotriene receptor-2 genes are less common in UM [11,15]. EIF1AX mutation is associated with low metastatic risk, while SF3B1 mutation is mainly associated with late-onset metastasis [16,17].

## 2. Dysregulated Pathways and Molecules Involved in UM Tumorigenesis and Metastasis

**Figure 1:**
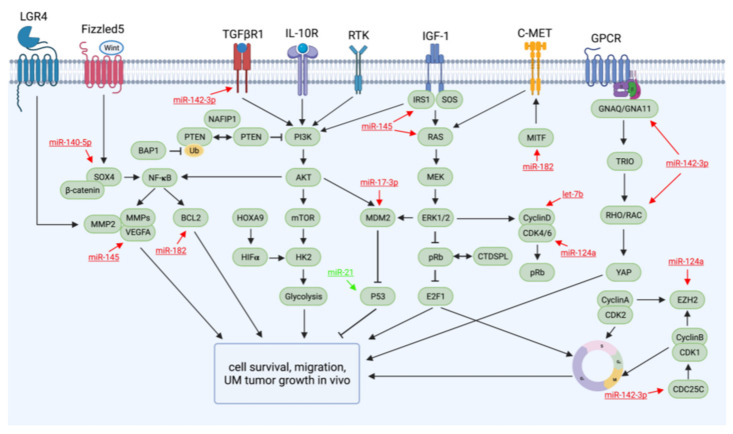
Major aberrant signaling pathways in UM and targets of miRNAs with therapeutic potential.

Particularly in UM, oncogenic GNAQ/11 mutations activate the RHO/RAC pathway by stimulating RAS homolog family member A and RAS-related C3 botulinum toxin substrate 1 (RAC1) [13]. Both mitogen-activated protein kinase/extracellular-signal-regulated kinase (MAPK/ERK) and phosphatidylinositol (4,5)-bisphosphate 3-kinase/protein kinase B (PI3K/AKT) pathways are dysregulated in response to GNAQ/11 mutations. ERK1/2 activation is critical for UM development [14], whereas phosphorylated AKT is associated with a high risk of metastatic disease [18]. In addition, oncogenic yes-associated protein and transcriptional co-activator with PDZ-binding motif dephosphorylation as a result of GNAQ/11 mutations is also essential for oncogenic activity in UM development [19] (Figure 1).

Some genes are highly overexpressed in primary UM and are functionally associated with pro-invasive properties of UM cells. For example, several matrix metalloproteinases (MMPs), such as MMP-2 and MMP-9, are highly expressed in primary UM and are correlated with a dismal prognosis; these MMPs mediate UM cell invasiveness [20,21,22]. The formation of micrometastases has been suggested to contribute to the invasiveness of UM and resistance to different treatments [23]. The hepatic microenvironment provides multiple growth and survival factors to UM cells, as well as several inflammatory and profibrogenic factors that are important in the homing of UM cells to the liver. For instance, UM liver metastases are associated with the strong expression of the tyrosine kinase receptor c-Met and hepatocyte growth factor (HGF, the ligand of c-Met, primarily produced in the liver) [24]. C-Met/HGF contribute to the activation of the PI3K/Akt pathway and promote the survival and pro-invasive activity of UM [25,26]. In addition, insulin-like growth factor 1 receptor is expressed in metastatic UM and promotes the proliferation of metastatic UM cells [27]. Of note, UM is highly vascularized, and vascular endothelial growth factor A (VEGF-A) is found in the aqueous humor and serum in patients with mUM [28]. Overexpression of VEGF-A is found in primary and metastatic UM cell lines, and VEGF-A signaling sustains the proliferation of UM cells [29].

Epigenetic modifications play crucial roles in gene regulation through altering DNA and histone structures, and also play critical pathogenic roles in UM [30]. The global methylation profile is associated with mutations of the *BAP1* gene that are associated with a distinct metastatic risk of UM [31]. Hypermethylation of the promoters of genes including Ras-association domain family 1 isoform A, p16 and *INK4a* is commonly observed in UM, thus suggesting the inactivation of these tumor suppressor genes in UM [32,33,34,35]. Moreover, phosphatase and TENsin homolog (PTEN), a tumor suppressor, are frequently under-expressed in UMs [36].

The aforementioned GNAQ/11 mutation-activated downstream signaling pathways and abnormally expressed molecules are actionable targets [37,38]. Molecular targeting may be one of the most promising therapies for UM treatment, and several studies have focused on targeting aberrant driver mutations and their downstream pathways in UM pathogenesis; these efforts have led to new therapeutic possibilities for UM treatments. Croce et al. [39] and Li et al. [40] have extensively reviewed the molecular targets in preclinical studies and summarized the ongoing clinical trials for UM. Notably, the inhibition of GNAQ/11 pathways with MEK inhibitors has been successful in some preclinical studies and clinical trials; however, none of these inhibitors have been found to increase the overall survival rate [39,41,42]. The interactions of GNAG/11-mediated downstream pathways may be responsible for the failure of single-target strategies, thus suggesting that UM therapies involving the simultaneous inhibition of different downstream pathways in combination may hold promise [42]. Unfortunately, the encouraging results of preclinical studies using combinational therapies co-targeting multiple pathways were not confirmed in early clinical studies [39]. Furthermore, several multikinase inhibitors that co-target multiple intracellular and cell surface kinases have been tested in advanced UM. These inhibitors include cabozantinib, which inhibits c-Met, AXL receptor tyrosine kinase and VEGF receptors (VEGFRs) [43], and sunitinib, which targets platelet-derived growth factor receptors, VEGFRs and CD117 (c-KIT) [44]. Early clinical studies have indicated the potential activity of cabozantinib or sunitinib in UM [43,45]. Combination trials with immunotherapy agents, histone deacetylase inhibitors and radioembolization are currently ongoing [46].

Although targeted combination therapy has made great progress, the current therapeutic approaches of targeted therapies have yielded very low response rates for mUM [25]. Therefore, exploring other avenues of potential dysregulation in UM is imperative. Noncoding microRNAs (miRNAs) have emerged as critical epigenetic regulators involved in the pathogenesis of UM [47]. For example, some miRNAs have been identified to affect the transcription and/or translation of many key genes and pathways that contribute to UM [48,49].

## 3. miRNAs with Therapeutic Potential for UM, Identified in Preclinical Studies

miRNAs are involved in the regulation of a variety of pathophysiological processes through degrading mRNAs or inhibiting the translation of target genes [50]. A single miRNA can target multiple genes, and a single gene can contain several miRNA response elements and be targeted by multiple miRNAs. This multi-target action of miRNAs makes them attractive tools for the development of anti-cancer therapies [51]. Aberrant miRNA expression is observed in UM, and the dysregulation of miRNA expression has been recognized as an epigenetic mechanism underlying UM tumorigenesis and metastasis. miRNAs may function as oncomiRs or tumor suppressors in UM. OncomiRs are generally upregulated in cancers, and typically target tumor suppressors and promote tumorigenesis. Inhibition of oncomiRs may significantly decrease tumor cell proliferation, survival and metastasis. In contrast, tumor suppressor miRNAs are defined by their properties of downregulating oncogenes, and they are often lost or under-expressed in cancer cells [52]. GNAQ/11 mutations and some components of downstream pathways are direct targets of tumor suppressor miRNAs. OncomiRs and tumor suppressor miRNAs in UM, as well as their expression and target genes, have been reviewed in previous publications [47,53].

A wide range of miRNAs have been presumed to be important in UM progression, and preclinical studies have validated several dysregulated miRNAs in UM as potential targets for inhibiting UM growth and metastatic progression (Figure 1). In this review, we summarize the miRNAs exhibiting therapeutic potential in preclinical studies (Figure 1).

miR-21 is one of the oncomiRs examined in functional studies using in vivo animal models. Overexpression of miR-21 promotes the proliferation, migration and invasion of primary and mUM cells. The p53 gene is a direct target of miR-21, and inactivation of p53 and its downstream LIM and SH3 protein 1 (LASP1) by miR-21 leads to more aggressive phenotypes of UM cells. Inhibition of miR-21 decreases in vivo tumor growth [54]. Thus, the influence of UM tumorigenesis and metastasis makes miR-21 a potential target for the development of novel therapeutic strategies.

Furthermore, the anti-UM properties of several tumor suppressor miRNAs have been investigated in functional studies and have shown strong inhibition of UM cell proliferation, migration and invasion, as well as in vivo tumor growth repression. Let-7b is downregulated in radioresistant UM cells. Let-7b overexpression leads to the inhibition of UM growth and an increase in the radiosensitivity of mUM cells, such as OCM1 and OM431, through the targeting of cyclin D1 expression [55]. miR-17-3p increases the transcriptional activity of p53 by downregulating the expression of the oncoprotein murine double-minute clone 2 (MDM2) [56], which mediates the proteasomal degradation of p53 through its E3 ligase activity [57]. In addition to p53, the retinoblastoma tumor suppressor protein (RB) is a key player in cell cycle progression. The canonical RB pathway consists of RB1, cyclin D1, cyclin-dependent kinase 4/6 (CDK4/6), p16 and the E2F family [58]. miR-124a exhibits strong anti-UM effects by targeting CDK4/6, cyclin D2 and enhancer of zeste homolog 2 (EZH2) [59]. miR-140-5p is downregulated in UM cells and tissues. The proto-oncogene *SOX4,* a crucial transcription factor of differentiation and progenitor development, is a direct target of miR-140-5p [60]. miR-140-5p’s downregulation of the SOX4-mediated Wnt/β-catenin and NF-κB signaling pathways substantially suppresses in vivo UM tumor growth [61]. miR-142-3p has been found to decrease UM cell proliferation and migration as well as inhibit UM tumor growth in a suprachoroidal xenograft model [48]. MiR-142-3p directly targets several genes associated with GNAQ/11 and downstream signaling pathways, including GNAQ, RAC1, transforming growth factor beta receptor 1 (TGFβR1), cell division cycle 25C (CDC25C) and Wiskott–Aldrich syndrome protein (WASL) [48]. miR-145 directly targets not only oncogene insulin receptor substrate-1 (*IRS-1*) but also neuroblastoma RAS viral oncogene homolog (N-RAS) and VEGF, thus significantly suppressing UM cell invasion, angiogenesis and tumor growth [62,63]. miR-182 targets multiple oncogenic genes, including microphthalmia-associated transcription factor (*MITF*), cyclin D2 and pro-apoptotic B-cell lymphoma 2 (*BCL2*) [64]. MITF regulates the expression of the *c-Met* gene [65], which is overexpressed in more than 60% of UM and is associated with tumor aggressiveness as well as metastasis [66]. Thus, miR-182 interferes with the c-Met signaling pathway through the downregulation of MITF. Additionally, miR-182 participates in the tumor suppression network of p53 in UM [64].

The miRNAs with therapeutic potential listed in Table 1 are only a few of the many miRNAs reported to be involved in the tumorigenic and metastatic pathways of UM [47]. Notably, Aughton et al. have revealed that miR-181a was the only downregulated miRNA among three studies of miRNA expression in large, clinically well-defined UM samples [53]. The tumor suppressor role of miR-181a in retinoblastoma has been demonstrated in our previous studies [67].

## 4. Approaches of Therapeutic Targeting of miRNAs and Limitations of miRNAs in Translational Therapeutics

To restore tumor suppressors that are downregulated or deleted in cancer cells, administration of miRNA mimics (synthetic oligonucleotides) can re-establish miRNA levels to their basal non-pathological states and restore their biological functions [68]. Nonetheless, to inhibit oncogenes, several approaches can be used, such as antisense oligonucleotides (antimiRs), miRNA sponges and genetic knockouts. AntimiRs, including locked nucleic acid oligonucleotides (LNAs), have recently shown high-affinity targeting and inhibition of oncogenic miRNAs [69]. miRNA sponge vectors for the expression of transcripts with miRNA binding sites complementary to the targeted miRNAs have been used to sequester endogenous miRNAs and prevent their binding to target mRNAs [70]. Clustered regularly interspaced short palindromic repeat/CRISPR-associated protein 9 (CRISPR/Cas9) genome-editing technology has also been used as a potent genetic engineering tool to achieve miRNA loss of function [71].

Despite their therapeutic potential, miRNAs often function by targeting multiple genes, thus making them attractive for anti-tumor therapy but also risky because of their potential adverse effects on healthy tissues. In addition, the therapeutic development of therapeutic miRNAs (miRNA mimics or inhibitors) also has the drawbacks of low stability, low endocytosis and immunotoxicity. Because naked miRNAs and antimiRs can be rapidly degraded by nucleases in the serum and rapidly cleared by renal infiltration, they show poor penetration and are unable to diffuse spontaneously into cancer cells; however, intracellular localization is required for their therapeutic effects [72]. To overcome the limitations of therapeutic miRNAs, several strategies have been used. In addition to chemical modifications, such as phosphodiester or phosphorothioate internucleotide linkages, and the synthesis of LNAs, various nanotechnology-based systems have been developed and investigated to encapsulate therapeutic miRNAs within functionalized nanocarriers [73].

## 5. Nanotechnology-Based miRNA Delivery Systems

### 5.1. Nanodelivery Systems for miRNA Therapeutics

Several viral and non-viral miRNA delivery systems have been developed and demonstrated to protect therapeutic miRNAs against degradation, endosomal escape, cellular uptake and specific targeting [67,74,75]. Viral vectors including lentiviruses, adenoviruses, retroviruses, adeno-associated viruses and virus-like nanoparticles have been shown to successfully deliver transgenes encoding miRNA mimics or antagonists [76]. Despite their high infection efficiency and persistent transgene expression, viral vectors have the drawbacks of toxicity, inherent immunogenicity, potential triggering of oncogenic transformation and manufacturing complexity. Non-viral nanoparticles (NPs) have various advantages over viral vectors, owing to their low immunogenicity, biocompatibility, ease of production, controlled composition, ease of surface modification for targeted delivery and ability to deliver multiple therapeutic molecules with synergistic effects in one platform [73,77]. Numerous non-viral NPs are classified into inorganic, organic and hybrid NPs on the basis of their nanomaterials. Hybrid NPs are made of two or more types of nanomaterials and generally comprise a metallic or polymeric core covered by one or more lipid layers. Tyagi et al. [78] and Attia et al. [77] have described the advantages and drawbacks of non-viral NP delivery systems.

Inorganic NPs are derived from metals (e.g., gold, silver, carbon dots, rare-earth-doped semiconductors, quantum dots, iron-oxide or silica). They provide several advantages, including a unique and tunable size, shape-dependent optical properties and multifunctional capabilities [79]. Gold nanoparticles (GNPs) have received substantial interest over the past few years because they are easy to prepare and modify. They can be functionalized with thiol groups to increase their bonding to miRNA or a polyethylene glycol (PEG) layer to stabilize GNP nanostructures by limiting their aggregation and miRNA degradation; in addition, they can target specific ligands on the surface to bind target sites [80,81]. Furthermore, mesoporous silica NPs, a group of inorganic NPs, provide large active surfaces, enabling the attachment of various functional groups for targeted miRNA delivery [82]. Organic NPs include polymers, dendrimers, liposomes, micelles and solid lipid NPs (SLNPs).

(1) Polymers are macromolecules consisting of a long-chain backbone of smaller repeating units and side groups. Various types of natural and synthetic polymers have been applied in miRNA-based therapies. For instance, chitosan, a natural cationic polymer with strong binding affinity for nucleic acids at low pH, has enabled successful delivery of miRNA to multiple myeloma [83]. Because of its affinity toward conjunctival and corneal surfaces, chitosan can penetrate into the eye [84]. Additionally, polyethyleneimine (PEI) and polylactide-co-glycolide (PLGA) are important synthetic polymers. PEI-based NPs are the most commonly used polymeric NPs for gene delivery because of their high cationic charge density potential [85]. Studies have reported successful miRNA delivery with PEI-based NPs, thus resulting in significant anti-cancer effects [86,87]. Similarly, PLGA-based NPs have been used to deliver miRNAs into several different types of cancer cells, and have exhibited high transfection efficiency and relatively low cytotoxicity. Notably, PLGA is one of the few polymers approved by the US Food and Drug Administration for human administration, owing to its biodegradable and biocompatible properties [88]. Interestingly, intravitreally injected PLGA NPs can pass through the retinal layers and reach the retinal pigment epithelium; therefore, PLGA NPs can be used to encapsulate therapeutic miRNAs for treating posterior segment diseases such as UM [89,90]. A recent study has suggested that the polymer poly (N-isopropylacrylamide) (PNIPAM) has strong potential for UM treatment, because high concentrations of PNIPAM have been detected in the uveal tissue after systemic injection [91].

(2) Dendrimers are synthetic polymeric macromolecules consisting of multiple highly branched monomers, with high drug-loading capacity through either encapsulation or conjugation [78]. Notably, polyamidoamine (PAMAM) is one of the first dendrimer families to be fully characterized, synthesized and commercialized. PAMAM dendrimers are one of the smallest nanomolecules with a particularly precise molecular weight; high-generation PAMAM dendrimers have shown higher transfection efficiency and improved miRNA effects in several human cancer models [92,93].

(3) Liposomes are small, spherical artificial vesicles composed of an aqueous inner compartment surrounded by a lipid bilayer. They can be created from cholesterol and natural non-toxic phospholipids [94]. Several cationic liposomes have been developed for the efficient delivery of miRNAs to the target tumor tissues [95,96,97].

(4) Micelles are self-assembled amphiphilic particles composed of a lipid monolayer with a hydrophobic core and hydrophilic surface. They are easy to prepare, and show low toxicity and good tissue penetration [78]. Combination therapy with gemcitabine-conjugated micelles loaded with miR-205 has shown significant inhibitory effects on advanced pancreatic cancer [98].

(5) SLNPs are submicron-sized lipid emulsions with solid lipids. They have unique properties such as a large surface area, high drug-loading capacity and interaction of phases at the interfaces [99]. SLNPs used to encapsulate miRNAs are usually composed of the cationic lipids N-[1-(2,3-dioleyloxy)propyl]-N,N,N-trimethylammonium chloride (DOTMA) or 1,2-dioleoyl-3-trimethylammonium-propane (DOTAP)), neutral lipids (e.g., cholesterol and dioleoylphosphatidyl ethanolamine (DOPE)) and PEG [100]. Cationic lipids facilitate interaction with the cell membrane, thereby improving transfection efficiency. We have demonstrated that miR-181a-loaded SLNPs exhibit high inhibitory effects on retinoblastoma cell viability, and the co-incorporation of miR-181a and a chemotherapeutic drug (melphalan) into SLNPs exhibits a complementary anti-retinoblastoma effect [67].

(6) Other bio-nanostructures include bacterially derived nanocells (EnGeneIC Ltd., Sydney, Australia), a powerful NP drug delivery system for direct targeting and killing of cancer cells and simultaneously stimulating the natural anti-tumor immune response [101]. A recent study has shown that bacterial nanocells loaded with miR-34a strongly enhance the anti-tumor effects of TMZ in orthotopic glioblastoma xenografts [102]. In addition, exosome-mimetic NPs, which reproduce cell-derived exosome structures, physicochemical properties and loading capacity, have been demonstrated as another strategy for miRNA delivery [103].

In general, nanocarriers/NPs can improve drug effectiveness while decreasing systemic toxicity and improving pharmacokinetics in various ways, such as by encapsulating drugs in their cores, protecting drugs from early inactivation or biodegradation, controlling drug release and distribution, enhancing drug absorption by targeting cells, enabling specific drug delivery and delivering multiple therapeutic molecules for synergistic effects in a single platform [77].

### 5.2. The Developed Nanocarriers/NPs Relevant to the Potential Therapeutic miRNAs for UM

Various nanodelivery systems have already substantially influenced the development of miRNA therapeutics for cancer therapy. Only the successfully developed nanocarriers/NPs for miRNA delivery and potential therapeutic miRNAs for UM are listed here (Table 2).

To suppress the function of oncomiR miR-21, several studies have investigated the anti-cancer efficacy of the anti-miR-21 oligonucleotide loaded in (1) AS1411 anti-nucleolin aptamer-decorated PEGylated PLGA NPs, (2) acid-triggered charge-reversible graphene-based NPs with multilayer polymers, (3) GNPs and (4) chlorotoxin-coupled stable nucleic acid lipid NPs and three-way-junction (3WJ)-based RNA NPs for targeting various types of cancers [104,105,106,107,108]. Of note, the 3WJ core derived from packaging the RNA of the bacteriophage phi29 DNA packaging motor has been extensively studied to fabricate various RNA NPs [127].

To recover tumor suppression function, delivering mimics of downregulated tumor suppressor miRNAs into targeted cancer cells is the most commonly used approach. Maghsoudnia et al. [109] have encapsulated let-7b mimic in hyaluronic acid (HA)-coated generation G5 PAMAM dendrimers to target CD44 over-expressing non-small-cell lung cancer cells. In addition, cationic liposomes containing both miR-34a and let-7b have shown a powerful inhibitory effect on neuroblastoma [110]. Vencken et al. [111] have loaded miR-17 mimic into lipid–polymer hybrid NPs composed of PLGA and the cationic lipid DOTAP, and have revealed the efficient delivery of miR-17 into bronchial epithelial cells. miR-124a mimic encapsulated in rabies virus glycoprotein-labeled non-toxic disulfide-linked PEI NPs has been delivered into neuron cells [86]. The miR-142-3p mimic was loaded in G5 PAMAM dendrimers to target myeloid cells [112]. Because the tumor suppressor miR-145 inhibits multiple types of tumor cells, numerous nanocarriers have been developed for miR-145 mimic delivery [113,114,115,116,117,118,119,120,121], including micelles, protamine nanocapsules, PLGA NPs, redox-responsive chitosan-thiolated dextran NPs, magnetic NPs, polyarginine-disulfide-linked PEI NPs and GNPs. Additionally, miR-145 expression vectors carried by HA-PLGA/PEI NPs and chitosan polyplex NPs have been delivered into cancer cells and found to restore miR-145 expression levels [122,123]. To deliver miR-182, a hydrogel-embedded, PEGylated GNP has been synthesized and found to provide sustained release of miR-182 in metastatic breast cancer [124]. Interestingly, PEGylated GNPs with miR-182 can penetrate the blood–brain/blood–tumor barriers, reduce glioblastoma tumor burden and increase animal survival [125].

Notably, Rois et al. [126] published the first study using a GNP loaded mix of four tumor suppressor miRNAs mimics for UM treatment—miR-34a, miR-137, miR-144 and miR-182—which are downregulated in UM cells and have synergistic effects on UM cell viability. Remarkably, conjugation of the unique combination of miRNAs on GNPs has been found to overcome the limitations of these molecules and make UM cells more susceptible to chemotherapeutic SN38 (7-ethyl-10-hidroxycamptothecin, a topoisomerase I inhibitor).

### 5.3. Modification of NP Surfaces for Improving Biocompatibility and Active Targeting

**Figure 2:**
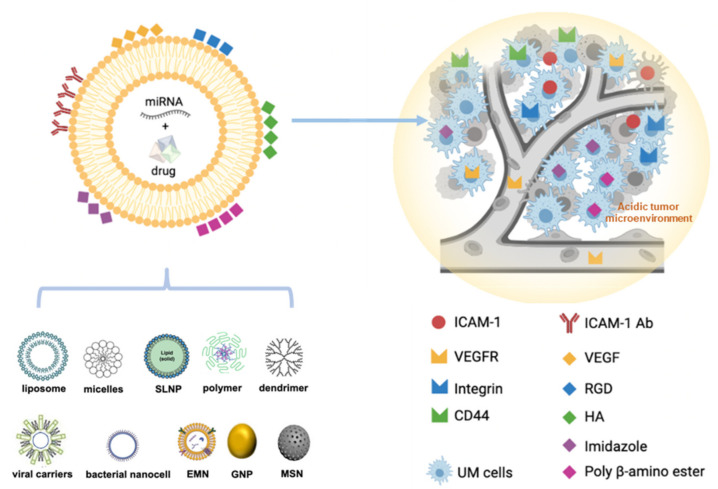
Schematic depiction of potential roles of NPs with surface modifications in UM treatment.

NPs are recognized as foreign bodies by the mononuclear phagocyte system (including monocytes, macrophages and Kupffer cells in the liver) and the complement system; thus, they are rapidly cleared from the blood [128]. Nevertheless, NP surface modifications can improve delivery efficacy and biodistribution. For instance, studies have shown that the PEG-based (DSPE-PEG2000) coating in NPs avoids clearance and improves stability in the blood [129,130,131,132]. Moreover, conjugating the surfaces of NPs with a specific ligand can significantly increase the quantity of drug delivered to the location of interest, thereby avoiding normal tissues, enhancing the therapeutic efficiency and limiting the adverse effects of the drugs [77,130]. In particular, UM cells strongly express the transmembrane glycoprotein intercellular adhesion molecule 1 (ICAM-1) and cell surface adhesion receptor CD44 [133]. ICAM-1-antibody-conjugated iron oxide NPs have been investigated for specific targeting of triple-negative breast cancer cells [134]. HA specifically binds the CD44 receptor and has been widely used in the synthesis of conjugated NPs for cancer-specific targeting [135,136]. UM develops in the choroid, one of the most capillary-rich tissues. Grafts of angiogenic factors such as VEGF or arginylglycylaspartic acid (RGD) peptides on inorganic NPs can be used to target tumoral angiogenesis [77]. Moreover, as a result of the active metabolism of tumor cells, the extracellular pH of tumor tissues is often acidic, owing to the accumulation of acidic metabolic waste products in the tumor microenvironment [137]. Therefore, pH-sensitive NPs have been developed for targeting the mildly acidic tumor microenvironment, such as polymers with imidazole groups or poly β-amino ester-based polymers responsive to tumoral low pH [138] (Figure 2).

## 6. Conclusions and Future Directions

With the increased knowledge regarding the dysregulation of miRNAs that underlie the oncogenesis of UM, preclinical studies of specific UM therapies are increasingly being reported. To achieve promising preclinical results and evaluate the effectiveness of potential therapeutic miRNAs, selecting orthotopic tumor models that create a disease-relevant environment and using cells or tissues from patients with UM (also known as patient-derived xenografts, or PDX), are important. Performing experiments on PDX models that reflect the heterogeneity and diversity of clinical tumors may decrease the dissimilarities between human tumors and preclinical models [130]. Notably, primary UM spheroids retain the histological and genetic characteristics of the primary tumor, and the use of 3D spheroids has enabled early phase drug screening [139]. Additionally, multicellular 3D models that recapitulate the spatial dimensions, cellular heterogeneity and molecular networks of the tumor microenvironment in vitro are excellent preclinical tools for exploring the roles of miRNAs in more clinically relevant settings.

Although the existing data are promising and support the utility of NPs as ideal carriers for miRNAs, several challenges remain before miRNA-based targeted NPs can be approved for clinical use, including tumor heterogeneity, penetration, endosomal escape, regulatory hurdles and the complex scaling up of the manufacturing process. Furthermore, for the successful design of targeted NP systems, ligand properties, target expression profiles and NP surface chemistry should be considered [78]. With continued improvements, the development of miRNA-based NPs with controllable/predictable biological identities may accelerate clinical translation.

Several delivery routes can be used for NPs to deliver miRNAs into the posterior segment of the eye for UM treatment [140]. These are systemic, periocular, suprachoroidal and intravitreal injection routes. The internal barriers include the blood–aqueous and blood–retina barriers, which impede direct and systemic drug access to the specific sites of action [141]. Therefore, systemic administration is not the preferable route for the treatment of UM, as the amount of drug that reaches the posterior segment of the eye is low and it is difficult to achieve an effective dose. The periocular injection route includes subconjunctival, sub-Tenon’s, retrobulbar, peribulbar and posterior juxtascleral injection [142]. Periocular injections are less invasive and capable of providing a relatively high drug bioavailability in the posterior cavity of the eye [143]. Intravitreal injection is becoming a more common choice for treating posterior eye diseases due to the possibility of offering a high drug load in the retina and vitreous and overcoming systemic exposure [144]. The drug molecular weight is the major factor affecting drug elimination for intravitreal injection. Besides, patients with posterior segment diseases usually need multiple intravitreal injections which may cause certain complications [140]. Notably, drug delivery into the suprachoroidal space (the potential space between the sclera and choroid) has emerged as a promising administration route with which to target multiple posterior eye diseases, including UM [145]. Studies have demonstrated the potential advantages of suprachoroidal drug delivery with NP-based gene therapy, in which therapeutic agents target chorioretinal tissues rather than the unaffected anterior section of the eye, thereby minimizing off-target effects [146,147,148]. In general, drug penetration ability and controlled release are the critical factors to maintain the effective therapeutic drug concentration in the posterior segment of eye. The small size of NPs can help to overcome the ocular barriers, and lipophilic NPs are more in favor of going through the blood–retinal barrier [149,150]. In addition, NPs can control drug release in a spatiotemporal manner to potentially enhance the therapeutic efficacy of the drugs, reduce toxicity and minimize the number of injections [151].

UM metastases are exceptionally hepatotropic. The hepatic microenvironment provides multiple growth and survival factors as well as inflammatory and profibrogenic mediators that are important in the homing of UM cells to the liver and mediates crosstalk between UM cells and hepatic stellate cells. Given that life-threatening micrometastases have usually already formed by the time of diagnosis, inhibiting the growth of these micrometastases is critical to confer major therapeutic effects. In this respect, better knowledge of the miRNAs involved in the metastatic microenvironment may provide new targets for UM therapy [7]. Regarding the evaluation of the anti-mUM efficacy of miRNA-based NP therapy, the development of orthotopic PDX models of mUM, particularly from high-risk primary UM or liver metastases, may have better clinical significance than ectopic PDX models [152]. In addition, the development of NPs with controlled biodistribution may improve the targeting of hepatic metastases [77]. Advanced NP delivery systems can deliver more than one therapeutic reagent, thereby enabling simultaneous targeting of several important oncogenic signaling pathways in UM to achieve better cytotoxic effects. Thus, the combination of conventional therapies with miRNA-based nanodelivery strategies may have potential for treating metastatic UM in the future.

## Figures and Tables

**Table 1:** List of miRNAs with therapeutic potential for UM, identified in preclinical studies.

miRNA	Preclinical Studies	Function	Target(s)	Ref.
miR-21-3p (oncomiR)	OCM-1 cells stably transfected with miR-21-3p inhibition vector s.c. injected into the right side of the axilla in nude mice	Reduces in vivo UM tumor growth	p53 and LASP1	[54]
Let-7b	OCM-1 cells stably overexpressing let-7b s.c. injected into the right flank in thymic nude mice	Increases radiosensitivity of UM cells	Cyclin D1	[55]
miR-17-3p	OCM-1A cells transfected with miR-17-3p agomir s.c. injected into the left axilla in nude mice	Suppresses tumorigenesis and metastasis of UM	MDM2	[56]
miR-124a	M23 cells or SP6.5 cells expressing miR-124a s.c. inoculated into the flank in nude mice	Suppresses UM tumor growth in vivo and inhibits UM cell invasion	CDK4/6, cyclin D2 and EZH2	[59]
miR-142-3p	miR-142-3p-transfected SP6.5 or M17 cells inoculated into the suprachoroidal space in nude mice	Inhibits cell proliferation, migration and invasion	CDC25C, TGFβR1, GNAQ, WASL and RAC1	[48]
miR-145	Lentivirus-miR-145-transduced OCM-1 cells s.c. injected in the right side of the axilla in nude mice	Reduces xenograft tumor growth and angiogenesis	IRS-1, N-RAS and VEGF	[63]
miR-182	M23 or SP6.5 cells expressing miR-182 s.c. inoculated into the flanks of nude mice	Suppresses in vivo UM growth	MITF, BCL2 and cyclin D2	[64]

**Table 2:** List of nanocarriers/NPs for the delivery of potential therapeutic miRNAs for UM.

Potential Therapeutic miRNA for UM	Nanocarriers/NPs	Targeting Cells	Ref.
Anti-miR-21 oligonucleotide	Aptamer-decorated PEGylated PLGA NPs	Ovarian cancers	[104]
	Acid-triggered charge-reversible graphene-based multilayer polymers	Triple-negative breast cancer	[105]
	GNPs	Breast cancer cells	[106]
	Chlorotoxin-coupled stable nucleic acid lipid NPs	Glioblastoma	[107]
	3WJ-based RNA NPs	Glioblastoma	[108]
Let-7b	HA-G5 PAMAM dendrimer	CD44^+^ non-small-cell lung cancer cells	[109]
	Cationic liposomes	Neuroblastoma	[110]
miR-17	DOTAP-modified PLGA lipid–polymer hybrid NPs	Bronchial epithelial cells	[111]
miR-124a	Disulfide-linked PEI NPs	Neuron cells	[86]
miR-142-3p	G5 PAMAM dendrimers	Myeloid cells	[112]
miR-145	Micelles	Vascular smooth muscle cells	[113]
	Disulfide cross-linked micelles	Colon cancer cells	[114]
	Nanocapsules	Colorectal cancer cells	[115]
	PLGA NPs	Vascular smooth muscle cells	[116]
	Chitosan-thiolated dextran NPs	Cancer cells	[117,118]
	Magnetic nanoformulation	Pancreatic cancer	[119]
	Polyarginine-disulfide-linked PEI NPs	Prostate cancer	[120]
	GNPs	Prostate and breast cancer cells	[121]
	HA-PLGA/PEI with miR-145 expression plasmid	Colon carcinoma	[122]
	Chitosan polyplex NPs with miR-145 expression plasmid	MCF-7	[123]
miR-182	PEGylated GNP nanogel	Metastatic breast cancer	[124]
	PEGylated GNPs	Glioblastoma	[125]
miR-34a, miR-137, miR-144 and miR-182	GNPs	UM cells	[126]

## Abbreviations

AKT	Protein kinase B
antimiRs	miRNA antisense oligonucleotides
BAP1	BRCA associated protein 1
BCL2	B-cell lymphoma 2
CDC25C	Cell division cycle 25 homolog c
CDK	Cyclin-dependent kinase
DOTAP	1,2-dioleoyl-3-trimethylammonium-propane
EIF1AX	Eukaryotic translation initiation factor 1A X-linked
ERK	Extracellular-signal-regulated kinase
EZH2	Enhancer of zeste homolog 2
GNAQ	Guanine nucleotide-binding protein alpha-Q
GNAQ/11	Guanine nucleotide-binding protein alpha-Q and subunit alpha-11
GNPs	Gold nanoparticles
HA	Hyaluronic acid
HGF	Hepatocyte growth factor
ICAM-1	Intercellular adhesion molecule 1
IRS-1	Insulin receptor substrate-1
LASP1	LIM and SH3 protein 1
LNA	Locked nucleic acid oligonucleotide
MAPK	Mitogen-activated protein kinase
MDM2	Murine double-minute clone 2 oncoprotein
miRNA	MicroRNA
MITF	Melanogenesis-associated transcription factor
MMPs	Matrix metalloproteinases
mUM	Metastatic uveal melanoma
NPs	Nanoparticles
PAMAM	Polyamidoamine
PDX	Patient-derived xenograft
PEG	Polyethylene glycol
PEI	Polyethyleneimine
PI3K	Phosphatidylinositol 3-kinase
PLGA	Polylactide-co-glycolide
PNIPAM	Poly N-isopropylacrylamide
PTEN	Phosphatase and tensin homolog
RAC1	ras-related c3 botulinum toxin substrate 1
RB	Retinoblastoma protein
RGD	Arginylglycylaspartic acid
SF3B1	Splicing factor 3b subunit 1
SLNPs	Solid lipid nanoparticles
TGFβR1	Transforming growth factor beta receptor 1
UM	Uveal melanoma
VEGF	Vascular endothelial growth factor
VEGFR	VEGF receptor
WASL	Wiskott–Aldrich-syndrome-like
